# Gastrointestinal In Vitro Digests of Infant Biscuits Formulated with Bovine Milk Proteins Positively Affect In Vitro Differentiation of Human Osteoblast-Like Cells

**DOI:** 10.3390/foods9101510

**Published:** 2020-10-21

**Authors:** Michela Bottani, Stefano Cattaneo, Valentina Pica, Milda Stuknytė, Marta Gomarasca, Giovanni Lombardi, Giuseppe Banfi, Ivano De Noni, Anita Ferraretto

**Affiliations:** 1 IRCCS Istituto Ortopedico Galeazzi, Laboratory of Experimental Biochemistry & Molecular Biology, Via Galeazzi 4, 20161 Milan, Italy; michela.bottani@grupposandonato.it (M.B.); marta.gomarasca@grupposandonato.it (M.G.); giovanni.lombardi@grupposandonato.it (G.L.); banfi.giuseppe@fondazionesanraffaele.it (G.B.); 2 Department of Food, Environmental and Nutritional Sciences, University of Milan, Via Celoria 2, 20133 Milan, Italy; stefano.cattaneo@unimi.it (S.C.); valentina.pica.94@gmail.com (V.P.); 3 Unitech COSPECT—University Technological Platforms Office, University of Milan, Via Golgi 19, 20133 Milan, Italy; milda.stuknyte@unimi.it; 4 Department of Athletics, Strength and Conditioning, Poznań University of Physical Education, Królowej Jadwigi 27/39, 61-871 Poznań, Poland; 5 Vita-Salute San Raffaele University, Via Olgettina 58, 20132 Milan, Italy; 6 Department of Biomedical Sciences for Health, University of Milan, Via Mangiagalli 31, 20133 Milan, Italy; anita.ferraretto@unimi.it

**Keywords:** infant biscuits, dairy proteins, whey, casein, Caco-2/HT-29 co-culture cells, Saos-2 cells, bone

## Abstract

Infant biscuits (IBs) are part of complementary feeding from weaning up to the age of five years. They normally contain bovine milk proteins, which can influence bone development. This potential effect was investigated using experimental baked IBs, which were prepared from doughs containing different type of dairy proteins: milk protein concentrate (IB1), whey protein isolate (IB2), and skimmed milk powder (IB3). Dairy protein-free (IB0) and gluten-free (IB4) biscuits were also formulated. The in vitro gastrointestinal digests of IBs (IBDs) were tested on a co-culture of Caco-2/HT-29 70/30 cells as an in vitro model of human small intestine. None of the IBDs influenced cell viability and monolayer integrity, while IBD0 and IBD4 increased Peptide-YY production. The basolateral contents of Transwell plates seeded with Caco-2/HT-29 70/30 co-culture, mimicking metabolized IBDs (MIBDs), were tested on Saos-2 cells, an in vitro model of human osteoblast-like cells. After incubation, MIBD0, lacking dairy proteins, decreased the cell viability, while MIBD2, containing whey protein isolate, increased both the viability and the number of cells. MIBD2 and MIBD4, the latter containing both casein and whey proteins, increased alkaline phosphatase activity, a bone differentiation marker. These results highlight that IBs containing dairy proteins positively affect bone development.

## 1. Introduction

The nutritional importance of milk and/or dairy products, even after weaning, is well known, since these foods contain components with a great bio-functional potential [1]. In particular, most of the literature highlights a positive correlation between bovine milk consumption, bone development, and health [2,3,4,5,6]. This effect depends on the presence of high-quality proteins together with bioavailable minerals [7]. Indeed, casein (CN) and whey proteins (WP) not only represent a fundamental source of amino acids but also play an anabolic role in bone formation [8]. CN is responsible for the insulin-like growth factor I secretion, which directly stimulates proliferation and differentiation of human osteoblasts [9]. Moreover, upon gastrointestinal digestion, CN releases phosphopeptides, which show the ability to bind calcium thus increasing its intestinal uptake and promoting bone mineralization [10,11,12]. CN also contains the amino acid proline that is required for the cross-linking of collagen fibers, involved in the bone matrix synthesis [13]. On the other hand, WP provide branched chain amino acids, above all leucine, able to activate mTORC1 signal complex. When active, mTORC1 triggers the post-meal synthesis of muscle proteins [14] necessary to support bone growth, induces insulin secretion (which exerts an anabolic role on protein synthesis) and suppresses bone resorption. The combination of the two milk protein fractions leads to positive effects on bone growth [15]. In addition to proteins, dairy foods contain nutrients—such as calcium, vitamin D, potassium, and phosphorus—all contributing to the bone health [7].

The “Global strategy for infant and young child feeding” by WHO and UNICEF established the necessity of providing an adequate amount and variety of foods to reach the right degree of growth and development in infants, especially during weaning and childhood [16]. Despite this, in Western countries, there is an increasing trend to exclude milk and/or dairy products from diet after weaning. In this regard, the Italian Guidelines for Healthy Eating report low levels of milk consumption during school age, a dietary habit probably associated to the introduction of complementary feeding of different sources [16,17]. Infant biscuits (IBs) are an important component of complementary feeding [16] and, therefore, they are formulated to fulfill infants specific nutritional demand. Nevertheless, the potential role of IBs formulated with bovine milk proteins in promoting bone development has not previously been investigated. In this regard, it is worth considering that the satiating property as well as the appetite regulation associated to dairy food consumption have been recently associated with the prevention and/or treatment of overweight and obesity, both conditions that negatively affect bone health and development [18,19,20]. Proteins are the nutrients with the most satiating properties [21], and dairy proteins were associated with the appetite and body weight control [22]. Both CN and WP are involved in the satiating power of dairy foods [23]. However, the duration of the effect is different due to the diverse gastrointestinal digestion rate of CN and WP [24]. Indeed, peptides and amino acids released from CN and WP during the gastrointestinal digestion can affect satiation by means of different pathways capable to modulate the secretion of Peptide-YY (PYY) and cholecystokinin (CCK) [23]. PYY is secreted by the enteroendocrine L-cells situated in the distal part of the small intestine, and its amount reaches a peak at approximately 2 h after a meal. CCK is secreted by the enteroendocrine I-cells situated in the first part of the small intestine, and its plasma level increases 15 min after a meal. These two hormones are able to induce satiety acting at the brain level and locally slowing down the gastric emptying [20]. On these bases, the present study was addressed to investigate the effects of five experimental IBs containing different dairy protein ingredients on bone development. To this purpose, IBs were digested in vitro and tested on a Caco-2/HT-29 co-culture [25], an in vitro cell model of human small intestine.

The effects of digests on intestinal barrier alterations and secretion of the anorectic hormones PYY and CCK were evaluated. After intestinal cell metabolism, the basolateral content of Transwell plates seeded with Caco-2/HT-29 co-culture (mimicking intestinal absorption) was tested in vitro on Saos-2 cells, a model of human osteoblast-like cells. The effects on viability, proliferation rate, and alkaline phosphatase (ALP) activity of Saos-2 cells were investigated. The final goal was to assess in vitro the potential role of the IB digests in promoting bone development.

## 2. Materials and Methods

### 2.1. Formulation of Experimental IBs

Five IBs (0, 1, 2, 3, 4) were prepared reproducing the gross composition and ingredients of commercial biscuits addressed for infant nutrition and sold in Italy. Sucrose, olive oil, and leavening agent (NaHCO_3_) were used for formulation all the IBs and were purchased at the local market. Soft wheat flour (Barilla, Parma, Italy) was substituted with wheat starch (Sigma-Aldrich, Milan, Italy) in formulating the gluten-free biscuits (IB4). Dairy powders included in the formulations of IB1 to IB4, from Fonterra Co-operative Group (Auckland, New Zealand), were skimmed milk powder (SMP), milk protein concentrate (MPC), and WP isolate (WPI). The composition of IBs is summarized in Table 1. Despite of the formulation, the total protein content of all the IBs was 5.9 g/100 g, whereas lactose was present (2.5%) only in IB3 (Table 2). The amounts of WP and CN in dairy ingredients were calculated on the basis of their total protein content (measured according to Standard ISO 8968-1:2014 [26]) and considering a CN-to-WP ratio 1:4, as it occurs in bovine milk. Lactose level in IB3 was calculated on the basis of lactose content in dairy ingredients, as determined according to Standard ISO 22662:2007 [27]. The gluten content of wheat flour was determined according to Standard ISO 21415-1:2006 [28]. All IBs were given a rectangular shape (about 8.0 × 2.5 × 0.5 cm) and were cooked for 11 min at 225 °C in a Hobart HO 300E electric oven (National MFG CO, Lincoln, NE, USA).

### 2.2. In Vitro Gastrointestinal Digestion of IBs

The in vitro COST-Infogest static gastrointestinal digestion protocol [29] was adopted with some modifications to simulate the gastrointestinal conditions of 6–12 months old infants [30,31]. All enzymes were purchased from Merck (St. Louis, MO, USA). Briefly, 5 g of minced IBs were mixed with 5 mL of simulated salivary fluid. Then, simulated gastric fluid (5 mL) together with porcine pepsin (800 U/mL gastric content) were added and incubated for 2 h at 37 °C, pH 3.0 (1 M HCl). For the intestinal phase, 2 mM of bile salts were dissolved in 10 mL of simulated intestinal fluid and added to each IB gastric digest. Intestinal enzymes were porcine trypsin (100 U/mL intestinal content) and bovine chymotrypsin (7 U/mL intestinal content), pancreatic amylase (50 U/mL intestinal content), intestinal lipase (130 U/mL intestinal content), and co-lipase (molar ratio lipase/co-lipase 2:1). After 2 h incubation at 37 °C, pH 7.0, digestion was stopped by adding the protease inhibitor AESFB (Roche, Mannheim, Germany) to achieve 1 mM final concentration. The digests were subsequently frozen at −24 °C.

At the end of the simulated static gastrointestinal digestion in vitro, all IBDs were adjusted, if necessary, for their osmolality to the physiological value of 300 mOsm/kg H_2_O (Osmometer basic, Löser Messtechnik, Berlin, Germany). For simplicity, the IB digests were named IBDs followed by their own corresponding number reported in Table 1. A possible effect induced by the simulated digestion fluids and enzymes (DF) was considered in each assay by adding to different cell wells a DF volume corresponding to that present in IBDs.

### 2.3. Human In Vitro Intestinal and Osteoblast-Like Cells

All cell culture media and reagents were purchased from Merck (St. Louis, MO, USA), while fetal bovine serum was from EuroClone Ltd. (West Yorkshire, UK). The parental cell lines HT-29 (BS TCL 132) and Caco-2 (BS TCL 87) were purchased from Istituto Zooprofilattico Sperimentale di Brescia (Brescia, Italy). Both cell lines were grown and differentiated separately according to Ferraretto et al. and Hekmati et al. [32,33]. The 70% Caco-2/30% HT-29 co-culture at the sixth day of post confluence (from here on named intestinal co-culture) was used as an in vitro model of human small intestine, due to its ability to mimic the morphological features of mucus secreting cells and enterocytes and the digestive/absorptive functions of this tract of human intestine [25]. All together, these features allowed to consider the co-culture a more versatile and complete in vitro human intestine model from the point of view of permeability and brush border enzyme digestion compared to the use of the parental cell lines Caco-2 and HT-29 in separate growth systems [25]. In fact, the 70% Caco-2/30% HT-29 co-culture at the sixth day of post confluence has been previously used to obtain the absorbable fractions from the in vitro digestion of different food matrix such as Grana Padano cheese [34] and gluten peptides [35].

Saos-2 cells (Saos-2 HTB-85, ATCC, LGC standards, Sesto San Giovanni, Italy) were used as a model of in vitro human osteoblast-like cells, and the protocol for their growth was applied as previously published [36].

### 2.4. Effects of IBDs on Intestinal Co-Culture Cell Viability and Monolayer Integrity

Each IBs digest was tested on co-culture using an appropriate volume of digests containing 50, 90, or 150 µg of initial IB/cm^2^ of intestinal surface area. The MTT cell viability assay [37], based on the mitochondrial metabolic activity, was performed on intestinal co-culture, seeded at 40,000 cells/cm^2^ in 96 multiwell plates, after reaching 80% of confluence in order to evaluate whether the selected IBs concentrations can affect cell viability. After 2 h at 37 °C of intestinal co-culture incubation with IBDs, cells were incubated with MTT (5 mg/mL) for the following 4 h. The formazan salt was solubilized in DMSO, and the absorbance was read at 570 nm with a Wallac Victor2 1420 Multilabel Counter plate reader (Perkin Elmer, Waltham, MA, USA). Results were expressed as percentage vs. the respective control of non-treated cells (CTR) and were obtained from three independent experiments, each of them consisting of at least two replicates.

The intestinal co-culture monolayer integrity was indirectly evaluated by TEER measurement (Millicell ERS system, Millipore Corporation, Billerica, MA, USA) on cells plated in a 24 well plate (Transwell Millicell^®^ Cell Culture Insert PET 1 µm, Millicell^®^ 24-Well Receiver Tray, Millipore Corporation) in their growth medium after 2 h incubation with IBDs. Measurement of TEER was done in three distinct regions of each well, then averaged and expressed as Ω cm^2^. The value of blanks, determined in absence of cells, were subtracted to all the values obtained in presence of cells. The final value reported on the graph, expressed as % vs. CTR (cells not incubated with IBDs), was obtained from two independent experiments each of them consisting of at least two replicates.

### 2.5. PYY and CCK Secretion Induced by IBDs

The IBDs were tested on intestinal co-culture cells seeded in a 24-well plate (Greiner bio-one Cellstar^®^, Milan, Italy) in 1 mL of RPMI. After 15 min or 2 h incubation, respectively, for CCK or PYY evaluation, the medium was collected, centrifuged to remove any presence of cells, and conserved at −20 °C until the ELISA assays, performed following manufacturer’s instructions (Labospace s.r.l., Milan, Italy). PYY production was expressed as pg/mL of collected medium, and results derived from a single experiment.

### 2.6. Effects of MIBDs on Osteoblast Viability and Cell Proliferation

For experiments with Saos-2 cells, IBDs were previously administered in 400 µL of HBSS to the apical chamber of 24 well plate (Transwell Millicell^®^) with co-culture cells plated on porous filters. After 2 h of incubation, the contents of the basolateral chambers, containing the metabolized IBDs (MIBDs), were recovered and tested on Saos-2 cells.

The Saos-2 cell viability was determined with the MTT assay [37], while the Trypan blue assay was performed to evaluate both the number of living cells (not colored and with intact membrane) and dead cells (blue-colored and with damaged membrane) [38]. In both cases, cells were seeded at 10,000 cells/cm^2^ in 96 and 24 well plates (Greiner bio-one), respectively, in their complete growth medium. The following day, 150 µg/cm^2^ of each MIBD was tested on the cells and incubated at 37 °C for three or seven days. At each time point the MTT assay was performed as described above, while in the case of trypan blue assay cells were detached from their growth support using 200 µL of trypsin-EDTA and, after detachment, 800 µL of their complete growth medium was added to stop trypsin activity. After trypan blue addition, the number of living (non-colored) cells was counted by means of a Bürker chamber, and results were expressed as a number (no.) of living cells/cm^2^ of growth area. Results were obtained from three independent experiments each of them consisting of at least two replicates.

### 2.7. Osteoblast ALP (EC 3.1.3.1) Assay

The assay is based on the formation of the colored p-nitrophenol (PNP) from the colorless p-nitrophenyl-phosphate (PNPP) [39]. The Saos-2 cells were seeded at 15,000 cells/cm^2^ in 6-well plate in their growth medium and, after 24 h, they were incubated with 150 µg/cm^2^ of each MIBD for seven days. At the end of the incubation period, cell medium was removed, an ice-cold physiological saline was added in each well, cells were detached with a cell scraper and collected in a centrifuge tube to be finally pelleted and store at −20 °C. The collected pellets were suspended in ice-cold Tris/Mannitol buffer (2 mM Tris, 50 mM mannitol, pH 7.1) to be homogenized and subsequently disrupted by ultrasonication (Sonoplus Ultraschall-Homogenisatoren, Bandeline, Germany), thus obtaining cell homogenate. PNP formation due to ALP activity was obtained incubating cell homogenate of each sample and CTR, for 25 min at 37 °C in the dark, with the reaction mixture (0.1 M NaHCO_3_, 5 mM MgCl_2_, and 7 mM PNPP). The reaction was stopped with 0.1 M NaOH, and the absorbance of samples, CTR and standards was read at 410 nm. The values were then converted in nmoles of PNP by means of a standard curve (from 10 to 100 nmoles PNP). The ALP specific activity was expressed as mU/mg protein (one ‘unit’ is defined as the enzyme activity able to hydrolyse 1 µmole substrate/min). The results were obtained from two independent experiments. Protein concentration was determined by Lowry method [40].

### 2.8. Statistical Analysis

For each experiment, the DF effect has been subtracted only when a statistically significant difference compared to the relative control was revealed. Statistically significant differences between mean values were established: (i) by one-way ANOVA followed by a Bonferroni post hoc *t* test with the SPSS 20 statistical software (SPSS, Chicago, IL, U.S.A.) to evaluate the effect of different concentrations of the same IB; (ii) by an independent two-sample *t* test in the case of IBDs and MIBDs vs. CTR and among two MIBDs. *p*-value < 0.05 was considered significant; differences between samples vs. CTR were represented by an asterisk, while differences among IBDs or MIBDs at different concentrations, when revealed, were marked with different letters.

## 3. Results and Discussion

### 3.1. In Vitro Effects of IBDs on Intestinal Cell Co-Culture

The inclusion of dairy ingredients in the formulation of IBs is mainly targeted to support the nutritional requirements of infants and, at the same time, to improve specific functionalities [41,42]. Considering the high consumption of IBs in infant and child population, our goal was to study experimental IBs formulated with ingredients useful for the development of the muscle-bone apparatus [43]. In this regard, the experimental IBs included in this study (except IB0) contained milk proteins such as WP alone (IB2) or in combination with CN (IB1, IB3, IB4) (Table 1). The presence of both CN and WP in IBs was obtained by including SMP (IB3) or MPC (IB1 and IB4) in the formulation, whereas the addition of WPI (IB2) allowed to supply only the WP fraction. Differently, the addition of MPC in IB4 was intended to totally replace gluten proteins in order to make a gluten-free IB. Overall, composition and type of dairy ingredients in the formulation reflected those of IBs mostly marketed in Italy. Being IB0 prepared without the addition of dairy ingredients, the protein fraction of this sample was represented only by gluten. The formulation of a dairy-free IB, as well as the inclusion of different dairy ingredients in experimental IBs, aimed to highlight the effects of the composition in vitro, especially the protein type, on intestinal and osteoblast-like cells.

The experimental IBs were digested according to an in vitro protocol suited for mimicking the gastrointestinal conditions of infants from 6 to 12 months. The international consensus gastrointestinal digestion in vitro method proposed by Brodkorb et al. [29] for adults was adjusted according to literature available so far regarding infant digestion [30,31]. Specifically, bile salt concentration and enzyme activities were reduced, while the composition of simulated gastric and intestinal fluids and time of digestion were the same as those adopted in the in vitro COST-Infogest digestion protocol for adults [29].

The volume of each IBDs tested on intestinal co-culture was determined by considering the amount of IBs usually consumed by infants (10, 20, and 35 g as a serving size) and their average intestinal absorbent area (24 m^2^) [44,45]. According to this, the serving sizes corresponded to about 50–90–150 µg of IBs/cm^2^ of intestinal surface area. For each in vitro experiment proper aliquots of IBDs to administer to intestinal co-culture were thus calculated considering the growth well area covered by cells.

**Figure 1:**
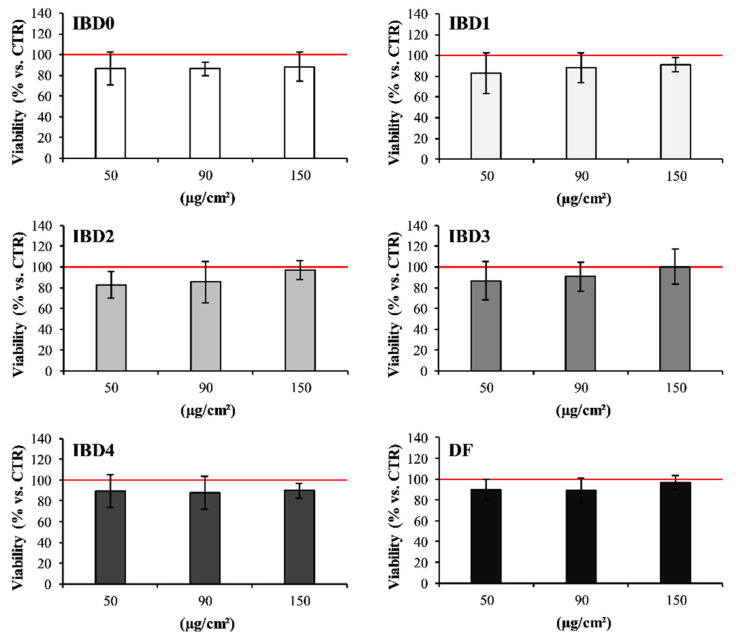
Intestinal 70% Caco-2/30% HT-29 co-culture viability after incubation with IBDs. The graphs in the figure represent the viability of intestinal co-culture cells after incubation with 50, 90, and 150 µg/cm^2^ of IBD0, IBD1, IBD2, IBD3, IBD4, and DF. The viability results are expressed as % vs. CTR (100), represented by the red line. Each % comes from the average of three independent experiments, each with at least two replicates.

**Figure 2:**
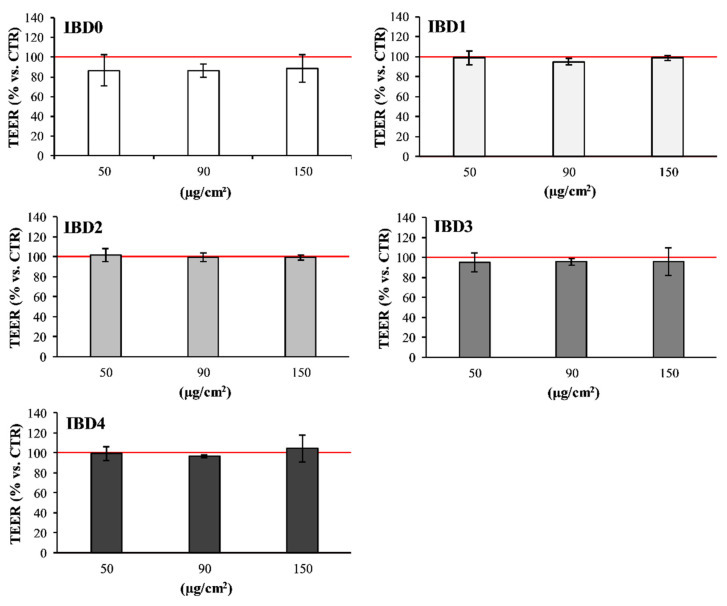
Intestinal 70% Caco-2/30% HT-29 co-culture TEER after incubation with IBDs. The graphs in the figure represent the monolayer integrity of intestinal co-culture cells after the incubation with 50, 90, and 150 µg/cm^2^ of IBD0, IBD1, IBD2, IBD3, and IBD4. The viability is expressed as % vs. CTR (100), represented as a red line. Each % comes from the average of two independent experiments, each with at least two replicates.

The effects of IBDs at the intestinal site were assessed by evaluating viability and TEER of the intestinal co-culture, since certain IB ingredients could promote intestinal barrier alterations or the onset of pathological conditions [46], which impair the correct bone development [47]. The administration of IBDs did not induce significant (vs. CTR) negative effects on intestinal co-culture cells under the adopted in vitro conditions. Indeed, IBDs did not affect either the viability of intestinal cells (Figure 1) or their TEER (Figure 2), despite the tested concentration.

**Figure 3:**
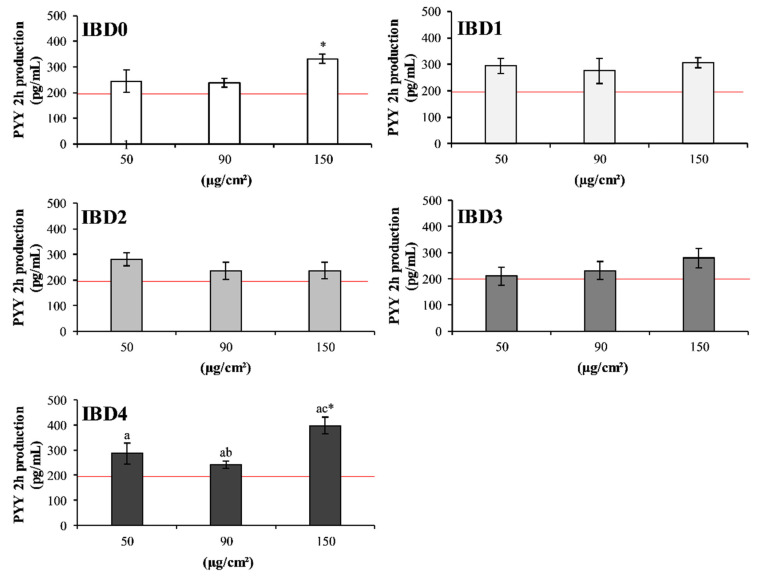
Differential secretion of PYY by intestinal co-culture after incubation with IBDs. The graphs in the figure represent the PYY production (pg/mL) of intestinal co-culture cells after 2 h incubation with 50, 90, and 150 µg/cm^2^ of IBD0, IBD1, IBD2, IBD3, and IBD4. Asterisks highlight statistically significant difference vs. CTR cells (red line, *p* < 0.05), while letters vs. different concentrations of the same IBD (*p* < 0.05). Each value comes from one independent experiment with two replicates.

The intestinal hormones CCK and PYY act as signals in the areas of the central nervous system involved in appetite inhibition. Both hormones are able to induce satiety even if in a different time range, thus preventing overweight and obesity, which in turn negatively affect bone health [18,20]. After a meal, the secretion of PYY and CCK is mainly due to the presence of proteins and related peptides released upon digestion in the intestinal lumen [23]. Indeed, dietary proteins are capable to increase PYY mRNA abundance and plasma concentration with a concomitant reduction of food intake [48]. Under this view, the satiating effects of IBDs could be related to different formulation and protein type of IBs [49]. The effects of the IBDs on the secretion of PYY by the intestinal co-culture are shown in Figure 3. The only significant (*p* < 0.05, vs. CTR) effect was revealed after 2 h incubation with IBD0 and IBD4, both at the highest tested concentration (Figure 3). From this point of view, the increased PYY secretion due to administration of IBD0 resembled the anorectic effects exerted by gluten in celiac patients. These patients show high circulating PYY levels, which return to standard values after the removal of gluten from their diet [49,50]. The increased PYY secretion in intestinal co-culture cells incubated with IB4 digest is in accordance with the well-recognized role exerted by the ingestion of milk proteins [51]. Indeed, IB4 contained the highest amount of dairy proteins (Table 2). Digestion of WP likely triggers PYY secretion due to the release and absorption of bioactive peptides and branched-chain amino acids, which improve secretion of incretin and GLP-1 as well. In this regard, leucine, mostly present in WP, acts at the hypothalamic level by regulating energy intake or expenditure [52]. In the case of CCK secretion, the incubation with IBDs revealed a dose dependent trend after 15 min, but without statistical significance (data not shown). Notwithstanding, this trend reflects the proteolysis occurring during IBs digestion, since amino acids and peptones (deriving from CN) are, among all other nutrients, the most potent stimulators of CCK secretion [24].

The CCK was not released from the intestinal co-culture here used probably due to several reasons. Although the co-culture comprises 70% Caco-2 cells, originally displaying an absorptive phenotype, it produces mucus [25] and PYY when it is grown in presence of an excess of nutrients [38], thus supporting its enteroendocrine capability. Nevertheless, the presence of PEPT1 transporter as well as of CaSR, both involved in CCK and GLP-1 secretion [53,54,55] has not been investigated so far. Indeed, it is reported that GluTag and NCI-H716 cell lines with enteroendocrine features do not release CCK [56,57], indicating that not all the enteroendocrine cells could secrete CCK. Another issue affecting CCK secretion is the nature of the protein hydrolyzates. Indeed, in CCK-secreting cell line STC-1 different sources of protein hydrolyzates were not able to affect CCK release but could stimulate the activation of the CCK1 receptor, CCK1R [58].

### 3.2. In Vitro Effects of MIBDs on Saos-2 Cell Culture

The use of intestinal co-culture cells, seeded on Transwell plates, allowed to complete the in vitro gastrointestinal digestion of IBs through the action of cell brush border peptidases. In addition, it allowed the IBDs to be metabolized and transferred through the intestinal epithelium into the basolateral chamber thus mimicking the passage of nutrients from the intestinal lumen to the blood stream. First of all, the possible effects of MIBDs on Saos-2 cell viability and proliferation were evaluated. Cell viability was measured by the MTT assay, which is an index of the cell metabolic activity due to the reduction of MTT by the glycolytic enzymes of the endoplasmic reticulum [59]. This assay can be used to monitor viability, toxicity, and cell stress even if cell death was detected; thus, it is an indirect measure of cell viability. Bone development was also investigated counting the numbers of Saos-2 cells, since an increased number of osteoblasts is linked to new bone deposition.

**Figure 4:**
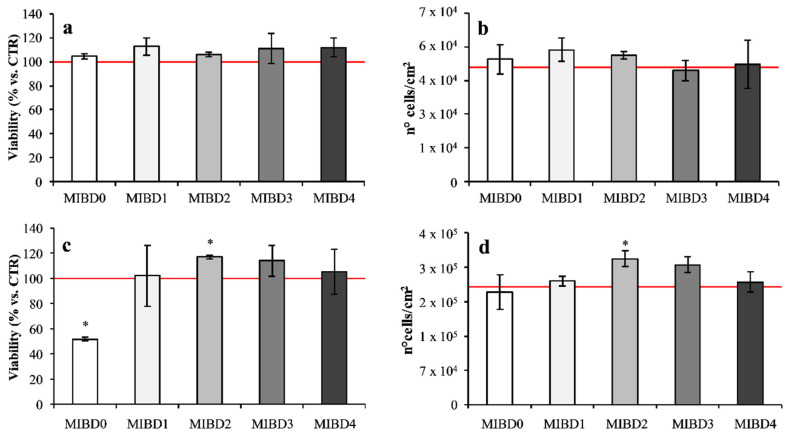
Viability and number of living human Saos-2 osteoblast-like cells after incubation with MIBDs. Viability and number of living cells were evaluated after three (**a**,**b**) and seven (**c**,**d**) days of cell incubation with 150 µg/cm^2^ of each MIBDs. Each bar represents the mean from three independent experiments, each with at least two replicates. Asterisks indicate statistical differences (*p* < 0.05) compared to CTR cells, indicated in the graph as a red line.

MIBDs affected osteoblast viability, proliferation and differentiation. Significant effects on viability were revealed (vs. CTR) after seven days of incubation with some MIBDs (Figure 4). Indeed, MIBD0 significantly (*p* < 0.01) halved the viability of Saos-2 cells vs. CTR cells, but maintained the cell proliferation rate. This result can be explained by considering that gluten likely supply the amino acids for maintaining the cell proliferation degree, but not the metabolic rate which is strongly impaired. In fact, the reduction of the viability was not monitored with MIBD4, lacking in gluten but containing the major quantity of CN and WP, and thus anabolic factors, such as leucine, and an amount of amino acids probably able to satisfy the energy demands for the metabolic activities in addition to their anabolic role. The specific ability exerted by WP on growth and differentiation of bone-forming cells was already reported in the scientific literature [60]. Our results suggest gluten is a suboptimal stimulus for bone development, but further investigations are needed to support this hypothesis. On the contrary, viability slightly increased with MIBD2 (*p* < 0.05), as assessed by the metabolic mitochondrial activity, as well as the increased number of living cells (*p* < 0.05), creating an optimal environment to improve bone synthesis. This effect is likely supported by the presence of WP-derived peptides, having IB2 the largest amount of WP (1.6 g/100 g), in accordance with previous in vitro data, reporting WP as anabolic factors for osteoblasts, modulating both their proliferation and differentiation [61] and favoring calcium deposition into the extracellular bone mineralized matrix [60]. Moreover, the content of certain amino acids (essential vs. non-essential), above all leucine, is higher in WP rather than in plant-derived proteins, and mostly concurs to bone development [62]. Administration of MIBD1, MIBD3, and MIBD4 revealed that CN together with WP did not affect the viability of Saos-2 cells and the number of living cells.

**Figure 5:**
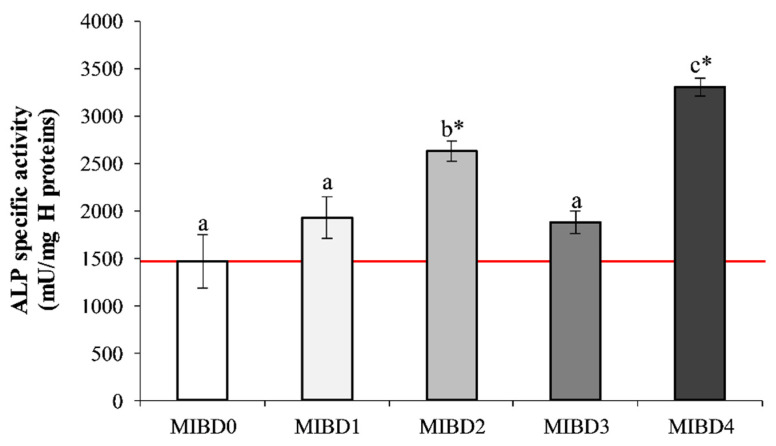
ALP activity of human Saos-2 osteoblast-like cells after incubation with in vitro MIBDs. ALP activity was measured after seven days’ incubation with 150 µg/cm^2^ MIBDs. Each bar represents the mean from two independent experiments. Asterisks indicate statistical differences (*p* < 0.05) from CTR cells, indicated in the graph as a red line, while different letters highlight significant differences among MIBDs.

The activity of ALP is currently monitored as an early differentiation marker of the osteoblast cell lineage [60]. As shown in Figure 5, the presence of WP alone or in combination with CN in IBs positively affected ALP activity. Indeed, the level of this marker significantly increased upon administration of 150 µg/cm^2^ of MIBD2 (79% increase vs. CTR, *p* < 0.05) and of MIBD4 (about 125% increase vs. CTR, *p* < 0.01) (Figure 5). The IB2 presented the highest WP content and, therefore, the corresponding digest was expected to positively affect osteoblast differentiation, based on the above reported signaling role. Contrarily to MIB2, MIB4 did not influence osteoblast proliferation, but induced a higher (*p* < 0.05) ALP activity. These metabolized samples were from IB2 and IB4, which contained the highest content of WP, and WP and CN, respectively (Table 2). This finding highlights that a large quantity of WP is needed to increase osteoblast proliferation. Nonetheless, the simultaneous presence of CN and WP is necessary to achieve the highest ALP activity and, consequently, the osteoblast differentiation [9,15].

## 4. Conclusions

The evidence presented here highlights the potential benefits of IBs in promoting osteoblast differentiation and, possibly, bone development. In this regard, the results revealed that the metabolized digests of IBs containing dairy proteins, especially WP, positively affect bone development by improving osteoblast viability, proliferation, and cell differentiation. Overall, these results give valuable information for formulating IBs to be used as complementary foods during weaning. Moreover, the same results might have some practical application for nutrition of infants presenting inadequate nutrient intake as a part of a complementary feeding diet. Nonetheless, many other factors influencing bone development have not been considered in the present study, and the long-term effects of this type of complementary feeding on later bone health need further investigations.

## Figures and Tables

**Table 1:** Composition (g/100 g) of experimental IBs. (MPC, milk protein concentrate; WPI, WP isolate; SMP, skimmed milk powder)

Ingredients		IB0	IB1	IB2	IB3	IB4
Wheat flour		58.9	43.0	43.0	43.0	0.0
Wheat starch		0.0	14.0	14.0	14.0	45.6
Sucrose		18.2	18.9	18.9	16.3	18.2
Olive oil		5.7	5.9	5.9	5.9	5.7
Dairy powders	MPC	0.0	1.9	0.0	0.0	7.4
	WPI	0.0	0.0	1.7	0.0	0.0
	SMP	0.0	0.0	0.0	4.7	0.0
Leavening agent		0.4	0.4	0.4	0.4	0.4
Water		16.8	15.8	15.8	15.8	22.9

**Table 2:** Type and quantity of protein and lactose content (g/100 g) of experimental IBs.

	IB0	IB1	IB2	IB3	IB4
Gluten	5.9	4.3	4.3	4.3	0.0
Casein	0.0	1.3	0.0	1.3	4.7
Whey proteins	0.0	0.3	1.6	0.3	1.2
Lactose	0.0	traces	0.0	2.5	traces
